# Quantitative contribution of iron-char composites mediating enhanced abatement on *p*-nitrophenol through extracellular electron transfer

**DOI:** 10.3389/fmicb.2026.1855444

**Published:** 2026-06-10

**Authors:** Jing Zhao, Xinyue Hu, Wangmin Wang, Anila Sikandar, Gang Chu, Wenxiu Qin, Kai Sun, Cheng Chen, Youbin Si

**Affiliations:** 1 Yunnan Provincial Key Laboratory of Soil Carbon Sequestration and Pollution Control, Yunnan International Joint Laboratory for Emission Reduction and Carbon Sequestration in Agricultural Soils, Faculty of Environmental Science & Engineering, Kunming University of Science & Technology, Kunming, Yunnan, China; 2 Faculty of Resources and Environment, Anhui Agricultural University, Hefei, Anhui, China

**Keywords:** electron transfer, Fe(II) dissolution, iron-char composite, microbial transformation, nitrophenol contaminant

## Abstract

The coupling application of carbonaceous material provides an innovative strategy to improve anaerobic bioreaction in wastewater treatment. This study synthesized biomass-waste-derived iron-char composites via a co-pyrolysis to explore the role on the biotransformation of *p*-nitrophenol (PNP) in anaerobic systems. With the increasing temperature, the proportion of crystalline magnetite was enhanced in iron-char composites. The transformation of Fe species from inorganic to organic combined states were evidenced by C − O − Fe bond formation as per XPS spectra, which improved the electron transfer performance of carbon matrix. Quantitative calculation demonstrated that iron-char composites significantly boosted microbial PNP degradation by 34.1–55.3%, especially for iron-chars of high temperature. The mediated electron transfer capacity of iron-char composites was a critical factor enhancing anaerobic PNP degradation with increasing pyrolysis temperature. In addition, the Fe(II) released from hematite/magnetite in iron-chars was observed, which was significantly responsible for PNP degradation. This study quantified the contributions of microbial metabolism, mediated electron transfer, and interfacial reactivity to microbial transformation of nitrophenol contaminant through establishing a structure-application relationship of iron-char composites. This work provides critical scientific insights and practical guidance for the bio-augmentation treatment of nitro-phenolic industrial wastewater.

## 1 Introduction

The nitrophenol contaminants are extensively detected in the wastewater from petrochemical, military, and pharmaceutical industries (Wang et al., 2023), which has been of great concern in wastewater treatment due to the toxicities and deleterious effects (Yue et al., 2018). The nitrophenol presence in water and soil through industrial discharge and agricultural irrigation leads to the ecological damage and health risk (Zhong et al., 2012). Great efforts thus have been devoted to eliminating nitrophenol compounds from wastewater. Multiple techniques such as sorption, filtration, advanced oxidation, aerobic/anaerobic bioreaction have been applied to remove nitrophenol contaminants (Dong et al., 2023; Karim and Gupta, 2003). *p*-Nitrophenol (PNP), one of representative nitrophenol contaminants, is an important fine chemical intermediate in pesticide, medicine, printing and rubber industries (Zhang et al., 2018). Owing to its recalcitrance, the PNP-containing wastewater causes severe environmental hazard when released into water system. Ahmed et al. (2021) concluded that the long-term exposure of PNP caused hepatotoxicity through apoptosis acceleration and glycogen accumulation in male Japanese quails. Even an exposure to low concentrations of PNP via ingestion can cause blood disorder or nervous damage in animals and humans (Bhatti et al., 2002). Thus, PNP monitoring in wastewater treatment is of great importance to guarantee renewable water security. The biotechnological application based on microorganisms coupled with carbonaceous materials is grabbing great attention in eliminating recalcitrant contaminants in wastewater treatment.

For microbial respiration, microorganisms evolve unique mechanisms to transfer electrons through conductive pili elongation, cytochrome expression advance, and polymeric substance secretion (Dong et al., 2020). For instance, the *Shewanella oneidensis* MR-1, belonging to the dissimilatory metal-reducing microorganisms, is documented that it possessed distinctive pathways to donate electrons to extracellular iron minerals (Shi et al., 2016). In engineering technology, the electron transfer capability of microorganisms has been harnessed for contaminant transformation. Song et al. (2024) reported a microbial facilitated advanced oxidation of PNP via boosting respiration metabolism by an electric field in simulated wetlands. Furthermore, the utilization of carbonaceous materials has been attempted to promote extracellular electron transfer for contaminant control in anaerobic bioreactor. Liu et al. (2020) highlighted the facilitation role of lignite on the microbial reduction of nitrobenzene by *Shewanella oneidensis* MR-1. Thus, the accelerating extracellular electron transfer from microorganisms could be a significant approach to achieve efficient removal of hazardous contaminants via coupling of bioreactor system with carbonaceous materials.

Biochar is a category of carbon-rich products through anaerobic pyrolysis of low-cost biomass wastes (Lehmann and Joseph, 2009). Apart from aromatic and aliphatic carbons, the various functional groups, multilevel pore structures, and several reactive species (e.g., persistent free radicals) are also included in biochar (Xiao et al., 2018). It has been widely applied for pollutant removal in wastewater treatment as well as soil remediation because of its sorption and degradation performance (Jin et al., 2016; Zhao et al., 2020). Biochar has been reported to possess significant electron transfer properties due to quinone/hydroquinone groups and graphitic carbon matrix (Wang et al., 2020; Sun et al., 2017), participating in an effective biotransformation of hazardous contaminants. Lei et al. (2022) considered biochar as an effective carbonaceous material for organic micropollutant removal in an anaerobic membrane bioreactor through the adsorption-biotransformation processes.

As the research progressed on functional biochar, several studies proposed that iron species were capable of boosting the reactivity and conductivity of carbonaceous materials (Chu et al., 2023). The iron species such as zero-valent iron or oxides could be incorporated in biochar through pyrolysis, loading, or precipitation. Yang et al. (2019) elaborated the mechanism to norfloxacin removal in wastewater by hematite- and pyrite-biochar composites, revealing that high interfacial adsorption and free radicals (∙OH and SO_4_^−^∙) generation were responsible for norfloxacin removal in non-biotransformation process. Zhuang et al., 2020 reported that a composite of Fe_3_O_4_/sludge-carbon enhanced the anaerobic bioreaction efficiency for azo dye wastewater via facilitating the interspecies electron transfer. The development of iron-char composite could be a promising alternative to improve extracellular electron transfer for contaminant removal in wastewater bioreaction (Yang et al., 2018). However, the green application of biomass-waste-derived iron-char composite in anaerobic bioreactor system is still lacking and the understanding of the contribution of iron-char composite in the transformation of nitrophenol contaminants in anaerobic wastewater treatment is limited, which significantly restrict the practical application.

Therefore, we selected a feasible and economical strategy for one-step co-pyrolysis of biomass wastes and iron oxides. This method is more convenient compared to others in operation (Tao et al., 2019; Yang et al., 2017; Li et al., 2024) and requires a lower iron amount (Supplementary Table S1) to synthesize iron-char composite for PNP removal in wastewater treatment. Moreover, the contribution of sorption and degradation of PNP by iron-char composite was evaluated during anaerobic biotransformation process. This work will provide an efficient and sustainable wastewater treatment strategy for nitrophenol removal.

## 2 Materials and methods

### 2.1 Preparation of iron-char composites

To compare the impacts of various components of biomass on structure evolution of iron-char composite, pine sawdust (PS), cellulose (CE), and lignin (LI) were purchased from Aladdin Co. for preparation. The purchased PS, CE, and LI were milled and sieved until the particles were less than 250 μm. These feedstock particles were fully mixed with hematite through ball-milling technique at the mass ratio of 5% Fe_2_O_3_ (≥99.99%). Mixed particles of PS, CE, LI were placed in a ceramic crucible into the muffle furnace device for the anaerobic co-pyrolysis. Nitrogen (100 mL/min N_2_) was firstly injected into furnace chamber for 30 min to evacuate oxygen (O_2_). The mixed particles were pyrolyzed at a temperature rate of 15 °C/min and then for 4 h at 350 and 650 °C. After the carbonization, the charring products were taken out from muffle furnace and crushed gently to particles with size less than 150 μm. Subsequently, the particles of all iron-char composites were placed into ziplock bags filled with N_2_, and noted as PFeX, CFeX, and LFeX (where X referred to the pyrolysis temperature).

### 2.2 Characterization of the iron-char composites

The elemental analyzer (Vario MicroCube, Elementar, Germany) was used to determine the elemental composition of the iron-char composites. The N_2_ adsorption instrument (Autosorb-1C, Quantachrome, United States) was used to determine the specific surface area (SSA) and micropore/mesopore volume. The iron oxide mineralogy of the iron-char composites was recorded by an X-ray diffractometer (EMPYREAN, Cu Kα, Netherlands) in the 2θ range of 5–65°. A vibrating sample magnetometer (VSM, 7407, Lakeshore, United States) was used to record the magnetization hysteresis loops of the iron-char composites at room temperature. The interfacial Fe species as well as the surface elements were analyzed by an X-ray photoelectron spectrometer (XPS, PHI5000, Versaprobe II, Ulvac-Phi, Japan). All Fe species spectra were deconvoluted using XPS peak 4.1 software. Electrochemical properties were evaluated using a three-electrode system of an electrochemical workstation (CHI660E, ChenHua, China). Electrochemical impedance spectroscopy (EIS) were recorded within the frequency range of 10^−2^–10^6^ Hz. The EIS curves were fitted using an equivalent circuit containing solution resistance (Rs), charge-transfer resistance (Rct), and a constant phase element. Tafel curves were recorded at a sweeping rate of 10 mV/s using a three-electrode system.

### 2.3 Microbial transformation

The *Shewanella putrefaciens* CN32 (*Sp.* CN32) was obtained from Guangdong Microbial Culture Collection Center and applied to microbial degradation of PNP with the iron-char composites in anaerobic condition. *Sp.* CN32 strains were inoculated to the solid medium of NB by the streak plate method and cultured at 30 °C for 24 h. The strains colonies were picked and subsequently cultured in the liquid medium at 30 °C for 24 h. After centrifugation at 2500 r/min for 10 min, *Sp.* CN32 strains were collected and washed 3 times with sterile phosphate buffer saline (PBS) (10 mmol/L, pH 7.0). The bacterium suspension was prepared with PBS solution in anaerobic glovebox for use within 2 h.

Microbial degradation experiment in anaerobic glovebox was conducted with 30 mL amber serum bottles that were treated by the autoclaved sterilization. All iron-char composites were weighed into serum bottles at the mass ratio of 1:1000 (solid/liquid mass). Subsequently, 29.7 mL of 50 mg/L PNP solution prepared with 0.01 mol/L deoxygenated PBS and 0.3 mL of *Sp.* CN32 suspension (at 0.8 of the optical density (OD600) value) was added to the reacting system. All experiments were conducted in triplicate in anoxic darkness condition for 168 h. One milliliter of the supernatant was extracted from the serum bottle at 12, 24, 36, 48, 72, 120, 168 h for the filtration of a 0.22 μm filter to detect PNP concentration. High performance liquid chromatograph (HPLC) (Agilent 1,200, Agilent Technologies, United States) with a C18 column was used to quantify PNP concentration at 316 nm. The miscible liquids of acetonitrile/deionized water (volume ratio of 40:60) served as mobile phase with flow rate at 1 mL/min.

To distinguish the sorption and degradation contribution, the solid-phase extraction using methanol (recovery efficiency > 92%) was carried out at 150 r/min for 12 h for accurate quantification of the interfacial residual concentration of PNP. The extraction procedure was performed in triplicate and extracted organic solution was collected for determination. The degraded amount of PNP was obtained based on the calculation of subtracting the sorption amount from the total amount. In addition, the cyclic experiments were done at 24 h intervals to observe the stability of iron-char composites in removal of phenolic compound. The procedure of three times cyclic experiments (72 h) is consistent with the above-mentioned method.

### 2.4 Iron dissolution

The dissolution kinetics of released iron including Fe(II) and Fe(III) ions from the iron-char composites were evaluated with continuous time. The iron-char composites were added to the reacting system containing microbes and non-microbes at the mass ratio of 1:1000 (solid/liquid mass) in the presence and absence of PNP, respectively. The released Fe(II) concentration was quantified by the o-phenanthroline assay method using an ultraviolet–visible spectrophotometer (Shimadzu, Japan). The total iron concentration of supernatant was digested by 10 mol/L nitric acid and then measured by the inductively coupled plasma mass spectrometry (ICP-MS, NexION 350x, PerkinElmer, United States).

## 3 Results and discussion

### 3.1 Sorption and degradation of PNP

**Figure 1:**
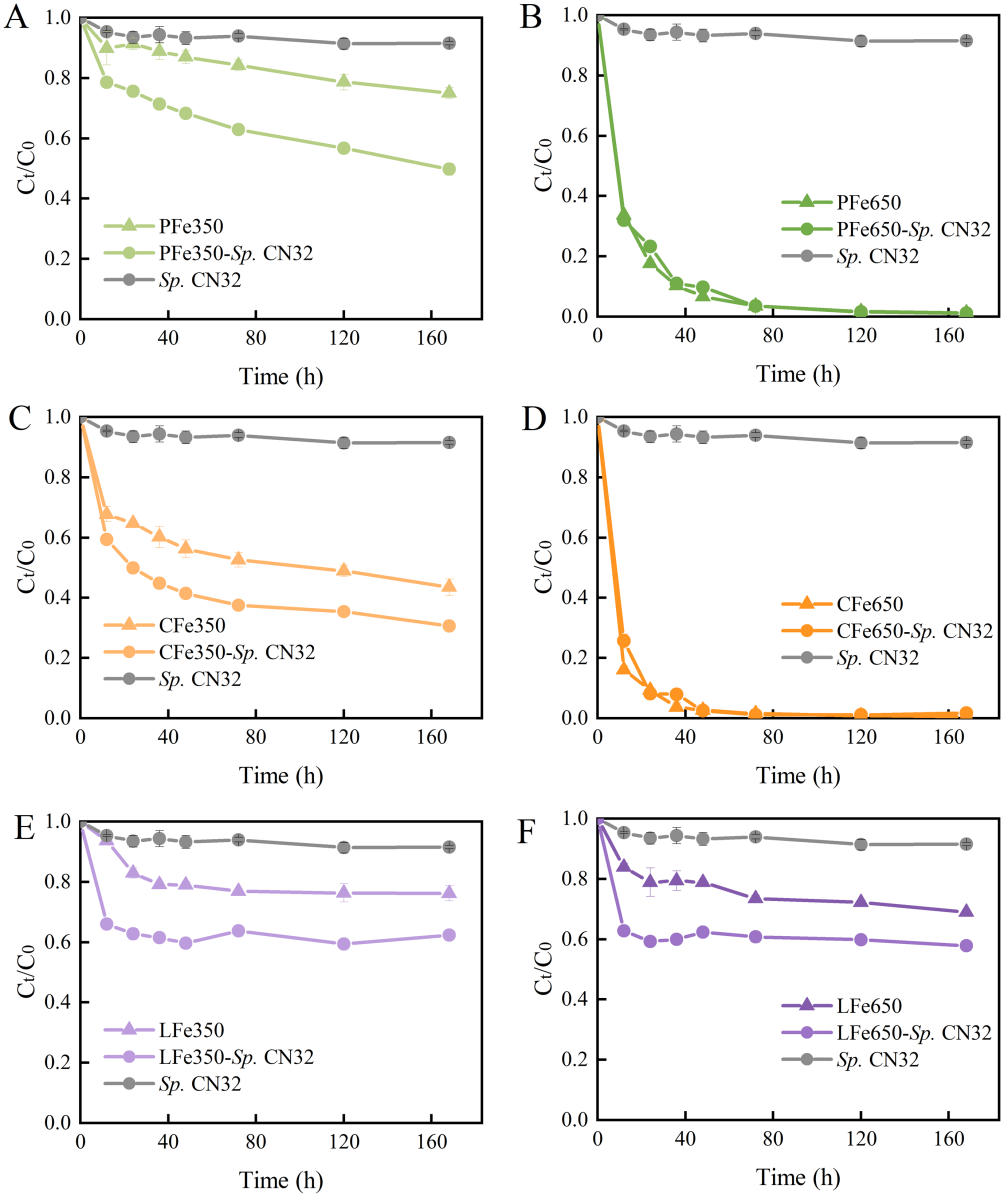
Removal kinetics of 50 mg/L PNP by PS, CE, LI-derived iron-char composites at 350 °C **(A,C,E)** and 650 °C **(B,D,F)** within and without *Sp.* CN32 system.

In the control experiment, *Sp.* CN32 exhibited limited PNP degradation in anaerobic environment in the absence of iron-char composites (Figure 1). Kinetics experiments were conducted with the iron-char composites for PNP removal for 168 h. Phenol removal without *Sp.* CN32 was observed in all iron-char composite cases. The kinetics curves were fitted with a two-compartment first order kinetics model (details in Supplementary Text S1), and exhibit a rapid decline at first and then flatten off. Especially for PFe650 and CFe650, the aqueous concentrations of PNP approached zero after 120 h. PNP concentrations did not show complete decline in other systems. This suggests that cellulose-derived iron-char composites of high-temperature could be an effective geological material to control nitrophenol contaminants. The curves were fitted with a dual-stage first order kinetics model. The model assumes that reacting sites of iron-char composites are not depleted and that nitrophenol reaction was predominantly dependent on the sorbed-state. Notably, the reacting rates of fast stage (*k*_1_,) were larger than those of slow stage (*k*_2_,) (Supplementary Table S2). The observed strong difference in reacting rates suggests that these kinetic curves could be interpreted by both sorption and degradation processes: (a) sorption is driven by the concentration gradient of PNP, and (b) degradation occurs due to the interfacial reactivity of iron-char composites (Li et al., 2020). After 72 h, the *k*_2_ values represented the slow rate of sorbed-state due to the exhaustion of interfacial reacting sites of iron-char composites.

**Figure 2:**
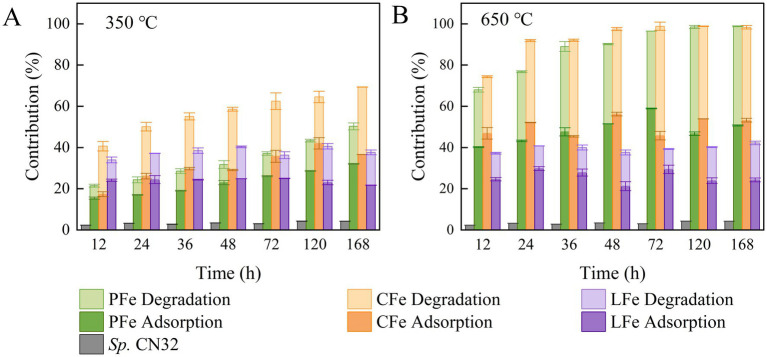
The contribution of adsorption and degradation of PNP by PS, CE, LI-derived iron-char composites at 350 °C **(A)** and 650 °C **(B)** in the presence of *Sp.* CN32 with the reaction time.

**Figure 3:**
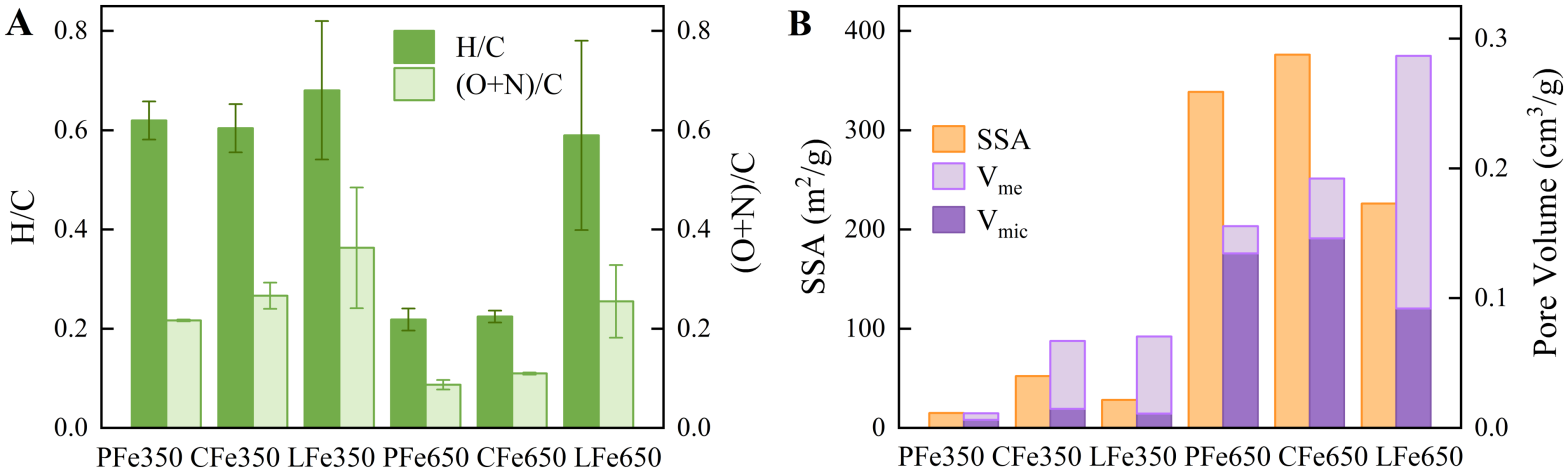
**(A)** The H/C (aromaticity) and (O + N)/C (polarity) and **(B)** SSA and pore volume of PS, CE, LI-derived iron-char composites at 350 °C and 650 °C.

Importantly, PNP removal was distinctly enhanced by *Sp.* CN32 in iron-char composite system, which was attributed to iron-char composite-mediated microbial transformation of nitrophenols. It was clear that the fraction of fast stage (*f*_1_) was increased and the fraction of slow stage (*f*_2_) was decreased (Supplementary Table S2). The *f*_1_/ *f*_2_ values were enlarged probably due to the iron-char composite-mediated microbial degradation. Thus, we conducted the solid-phase extraction experiment of iron-char composite system for distinguishing the contribution of sorption and degradation in the presence of *Sp.* CN32. Two phenomena were observed as shown in Figure 2. First, the cellulose-derived iron-chars exhibited higher sorption and degradation compared to the lignin-derived iron-chars at the same temperature. Compared to lignin-derived iron-chars, smaller H/C and (O + N)/C values represented that the cellulose-derived iron-chars possessed stronger aromaticity and lower polarity (Figure 3A). The thermal conversion of cellulose produced more condensed aromatic carbons and eliminated more O-containing groups. Liu et al. (2017) elucidated that cellulose can be depolymerized to oligosaccharides through the dehydration and then the charring of oligosaccharides occurred through the cyclization and condensation. This results in a higher microporosity and a larger SSA of the cellulose-derived iron-chars that promoted the sorption of PNP (Figure 3B). On the other hand, the electron transfer resistance (as indicated by Rct) of cellulose-derived iron-chars was distinctly smaller compared to lignin-derived iron-chars owing to its high aromaticity (Li et al., 2021). For instance, CFe350 and CFe650 had small Rct values of 6.68 *Ω* and 5.47 Ω acquired from electrochemical impedance, respectively. The result indicates a strong performance of electron transfer via carbonaceous matrix of cellulose-derived iron-chars, which is conducive to the mediated microbial degradation of PNP.

**Figure 4:**
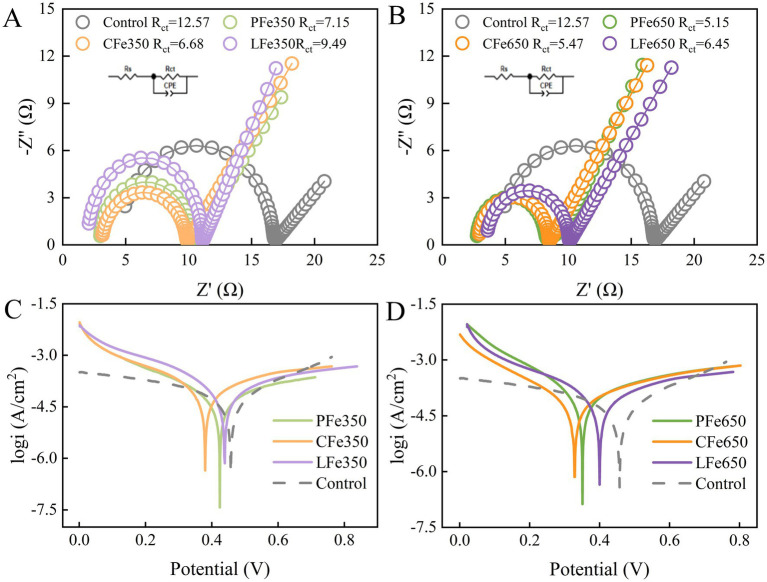
The EIS **(A,B)** and Tafel **(C,D)** curves of PS, CE, LI-derived iron-char composites at 350 °C and 650 °C; the control represents the experiment of no iron-char composite system.

Second, the iron-char composites of high temperature exhibited more degradation than those of low temperature in the presence of *Sp.* CN32 (Figure 2). One probably interpretation of this phenomenon is that sorption contributes more to the interfacial reaction for PNP. A reduction in H/C and O/C ratios was manifested in iron-char composites with increasing temperatures from 350 to 650 °C. Thermolytic elimination of organic volatiles fractions is responsible for the elemental change. This structural reorganization ultimately yielded a highly carbonized skeleton dominated by aromatic domains and a well-developed microporous network (Figure 3). The iron-char composites of high temperature were beneficial to the sorption of PNP, especially CFe650 and PFe650. Although PNP removal by PFe650 and CFe650 appeared to be nearly close in the absence and presence of *Sp.* CN32, the degradation contribution within microbial system was increased (Supplementary Figure S1). In EIS spectra, the diameter of the semicircular arc along the x-axis represents the charge transfer resistance (Rct) of each iron-char composite. The smaller Rct values were observed for the iron-char composites of high temperature (Figures 4A,B), suggesting that the *π*-conjugated system of aromatic carbons has an excellent capacity to facilitate the extracellular electron transfer during the metabolism of *Sp.* CN32. Tafel curves further demonstrated the electron transfer performance of the iron-char composites using the liner correlation between material potential and logarithmic current density (Figures 4C,D), which was in line with the EIS study. The redox rate at the surface of iron-char composites can be reflected by I_0_ values. The I_0_ values were calculated by extrapolating the slope of current density to zero overpotential through a linear regression between 60 and 120 mV (Yan et al., 2024). As shown in Supplementary Table S3, the I_0_ values followed the order: PFe650 > CFe650 > LFe650 > CFe350 > PFe350 > LFe350. It was clearly seen that both CFe650 and PFe650 exhibited an excellent potential to facilitate electron transfer through the carbonaceous interface. Thus, the microbial degradation to PNP molecules in the sorbed-state could be improved by the iron-char composites of high temperature.

### 3.2 Iron oxide conversion

**Figure 5:**
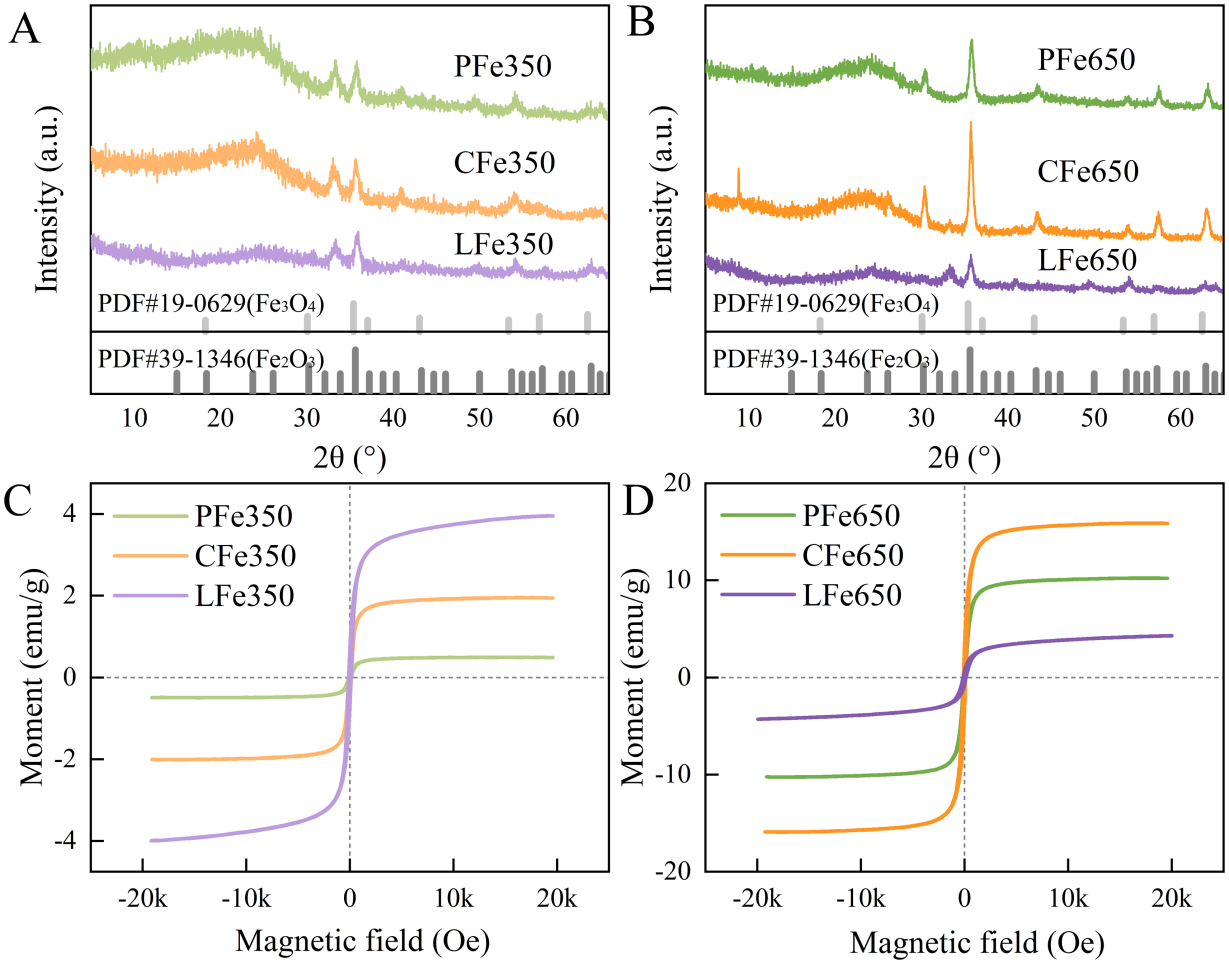
The XRD patterns **(A,B)** and magnetization hysteresis loops **(C,D)** of PS, CE, LI-derived iron-char composites at 350 °C and 650 °C.

X-ray diffraction (XRD) patterns provide the mineralogy changes of iron oxide. In iron-char composites, we observed the existence of magnetite from hematite transformation after the co-pyrolysis (Figures 5A,B). The diffraction peaks at 33.3°, 35.8° and 54.1° in iron-chars at 350 °C corresponded to the (310), (311) and (422) planes of hematite, respectively. With the rising temperature from 350 °C to 650 °C, the intensity of diffraction peak at 34°, especially for PFe650 and CFe650, was decreased significantly and even disappeared. But, four typical diffraction peaks at 30.4°, 43.4°, 57.5°, and 63.2° emerged and the signature peak at 35.8° was increased sharply, suggesting an enhancive proportion of crystalline Fe_3_O_4_ (JCPDS 00–019-0629) in carbon matrix. The conductivity of the iron-char composites was in close relation to the crystalline structure of magnetite. The formation of crystalline magnetite resulted in the enhanced conductivity of the iron-char composites of high temperature as supported by Rct values, which was conducive to extracellular electron transfer during the proliferation of *Sp.* CN32.

An excellent correlation was observed between crystalline magnetite and its magnetism of iron-chars, determined by XRD and magnetization hysteresis loops (Figures 5C,D). The saturation magnetization of PFe350, CFe350, LFe350 and PFe650, CFe650, LFe650 was 0.49, 1.95, 3.95 and 10.23, 15.86, 4.30 emu/g, respectively. It was clear that the magnetization of cellulose-derived iron-chars was significantly increased with the pyrolysis temperature from 350 to 650 °C, which was related to the evolution of crystalline magnetite. The charring of cellulose starts with the C − O bond depolymerization to produce CO simultaneously (Liu et al., 2017). A large amount of the reductive gases like CO contributes to the evolution of hematite to magnetite with pyrolytic temperatures. The iron-chars exhibited the increasing magnetism with the pyrolysis temperatures, which was attributed to progressive generation of magnetite. But, the magnetic properties of lignocellulose-derived iron-chars exhibited divergent phenomena at different temperatures. LFe350 showed the higher magnetism than CFe350. Lignin undergoes progressive cracking around 350 °C, promoting the generation of reducing gases that make iron-mineral transformation more thoroughly. In contrast, CFe650 exhibited the maximum magnetism among the iron-char composites probably due to extensive cellulose decomposition via gas-phase reduction pathway (Wei et al., 2020).

The magnetic property of iron-char composites guaranteed the separation in material application. Supplementary Figure S2 displays the cyclic utilization of iron-char composites through three cyclic experiments at 24 h intervals. The iron-char composites exhibited a sustainable cycle performance to PNP removal including sorption and degradation. Cyclic PNP removal by iron-chars remained in the range of 79.86–99.92%. Moreover, cellulose-derived iron-chars exhibited high-efficiency recycling rate. The cyclic efficiency of CFe350 achieved 99.92%, while that of CFe650 achieved 90.84% for PNP removal. The iron-char composites strongly exhibited magnetic separability and chemical stability, which supported their application in large-scale continuous wastewater treatment.

**Figure 6:**
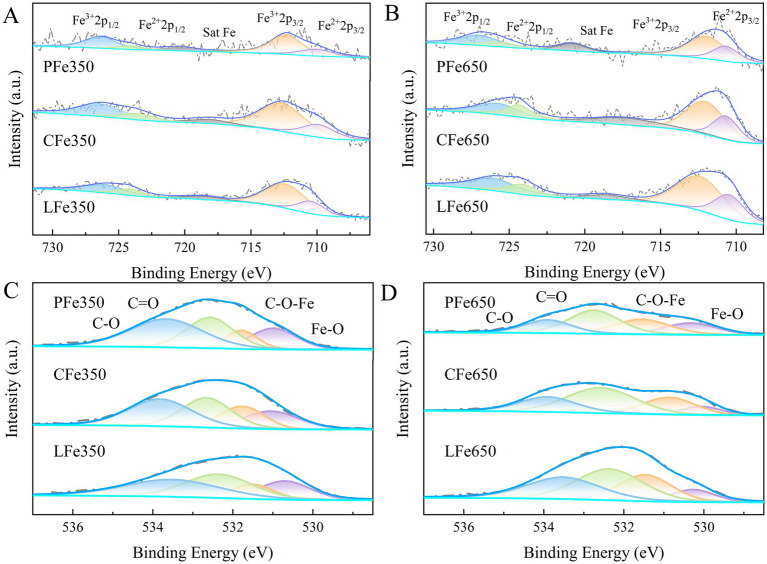
The XPS spectra of Fe 2p **(A,B)** and O 1 s **(C,D)** of the iron-char composites at 350 °C and 650 °C.

On the basis of XPS (Figures 6A,B), the 2p orbit of Fe was split into two peaks around at 723. 00 and 711.00 eV. Both peaks assigned to Fe 2p_1/2_ and Fe 2p_3/2_ through spectra deconvolution, which further confirmed the existence of distinct Fe species in carbon matrix. Fe 2p_1/2_ at 724.00 eV and Fe 2p_3/2_ at 710.50 eV attributed to Fe(II) peaks, whereas the intensities of Fe 2p_1/2_ at 726.00 eV and Fe 2p_3/2_ at 712.50 eV assigned to Fe(III) response. The peak ratios of Fe(II) 2p_1/2_ and Fe(II) 2p_3/2_ were in range of 6.47–11.06% and 14.55–20.88%, respectively (Supplementary Table S4). The satellite ranging from 717.10 to 719.90 eV also involved with Fe(III) and Fe(II) species. The existence of Fe(II) species further demonstrated a structural transformation from Fe(III) to Fe(II) in iron oxide during anaerobic co-pyrolysis. The extracellular electron favors migration via crystalline magnetite to achieve rapid electron transfer because of coexistence permutation of Fe(II) and Fe(III) in iron-char composites of high temperature (Kato et al., 2012). In contrast, electron transfer via hematite in carbon matrix requires to overcome higher energy barriers, leading to a relatively lower conductivity of low temperature iron-char composites. The enhanced conductivity at the carbonaceous interface triggered an enhanced microbial degradation via iron-char-mediated electron transfer process.

As the pyrolytic temperature was increased, the C1s and Fe 2p content on carbonaceous surface were enriched due to the elimination of O-containing groups (Supplementary Table S5). It was observed that the ratio of C − C/C=C bonds was promoted and of C − O bond was diminished in carbon matrix (Supplementary Figure S3; Supplementary Table S6). The XPS spectra of O1s revealed the presence of Fe − O, C − O − Fe, C=O, C − O, bonds of the iron-char composites (Figure 6; Supplementary Table S7). In anaerobic co-pyrolysis, the C − O/C=O bond was damaged owing to the generation of reductive gases (e.g., CO and H_2_) and volatile organic components (e.g., alkanes, hydrocarbons, and pneumatolytic tars). Furthermore, the lattice oxygen in hematite can be stimulated by pyrolysis temperature for the production of reactive Fe − O sites, generating C − O − Fe bond through the free radical reaction with volatiles during carbonization (Chen et al., 2019). The result implies that the co-pyrolysis of lignocellulose and hematite causes Fe transformation from inorganic to organic combined states. Yang et al. (2018) found a similar phenomenon that *α*-FeOOH nanocrystals were encapsulated in porous biochar framework via C − O − Fe bonding during the carbonization and the oxide-modified biochar possessed a highly effective capacity for copper removal.

### 3.3 Iron release

**Figure 7:**
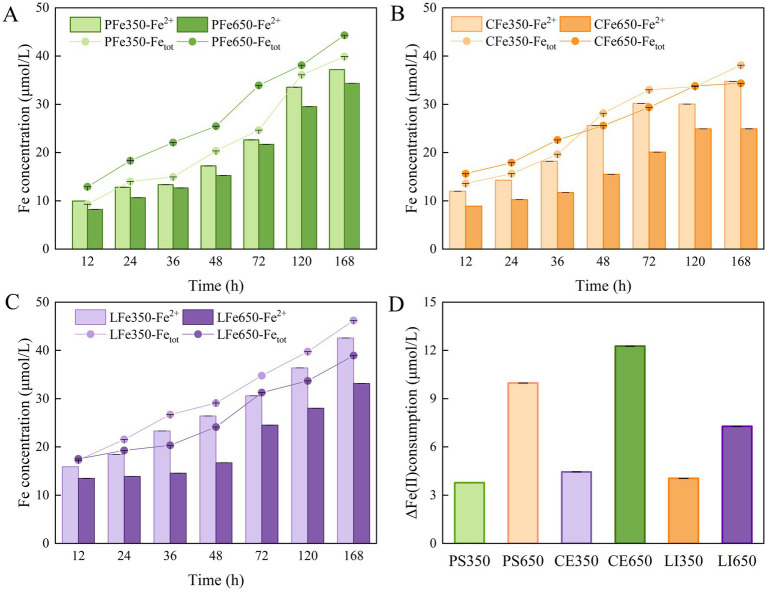
Iron dissolution kinetics including total Fe and Fe(II) ions of PS **(A)**, CE **(B)**, LI **(C)**-derived iron-char composites with *Sp.* CN32 and Fe(II) consumption to PNP reduction **(D)**.

The structural properties of iron-char composites are significantly related the dissolution and transformation of Fe(II) in anaerobic aqueous reactions, which is also closely associated with PNP degradation (Chu et al., 2023). Time-dependent iron dissolution kinetics is illustrated in Figure 7. Three phenomena were summarized. First, Fe(II) ions in aqueous phase were gradually released with the reaction time during microbial degradation by *Sp.* CN32, which was attributed to the dissimilatory reduction of iron oxides in carbon matrix. To verify the dissimilatory reduction process of hematite and magnetite, an experiment on the dissolution of Fe(II) ions was conducted at the same ratio of iron oxides (magnetite and hematite) with and without *Sp.* CN32 (Supplementary Figure S4). Supplementary Figure S4 shows that Fe(II) irons from magnetite and hematite were difficult to be released without *Sp.* CN32 system, whereas magnetite and hematite can release a large amount of Fe(II) irons within *Sp.* CN32 system and the released Fe(II) concentration was at a maximum for magnetite. Second, the amount of total iron dissolution was in accordance with released Fe(II) concentration (Figure 7). The concentration of total iron was larger than that of Fe(II) ions, implying a transformation of Fe(II) in anaerobic system. Third, the released amount of Fe(II) in the absence of PNP was larger than that in the presence of PNP (Supplementary Figure S5), revealing a consumption of Fe(II) to PNP degradation. Figure 7D displays the consumption amount of Fe(II) in anaerobic system. As for Fe(II) ions consumption for PNP reduction, CFe650, PFe650, and LFe650 exhibited a relatively high Fe(II) consumption, while CFe350, PFe350, and LFe350 showed a relatively low Fe(II) consumption. These results suggest that the iron-char composites of high temperature are more conducive to PNP degradation through released Fe(II) reduction from hematite or magnetite in the presence of *Sp.* CN32. The released Fe(II) made a significant contribution to PNP degradation via an abiotic reduction pathway in aqueous phase. This dynamic consumption of Fe(II) also offers information on electron transfer mechanisms of carbon matrix produced from various biomass components. The application of cellulose-derived iron-char composite exhibited superior ΔFe(II) consumption of PNP to that of lignin-derived iron-char composite, which was attributed to higher electron transfer efficiency of CFe350and CFe650, particularly for CFe650 system (Huo et al., 2025). Thus, cellulose component could be a promising feedstock to manufacture iron-char composites for phenolic wastewater treatment.

### 3.4 Degradation contribution

**Figure 8:**
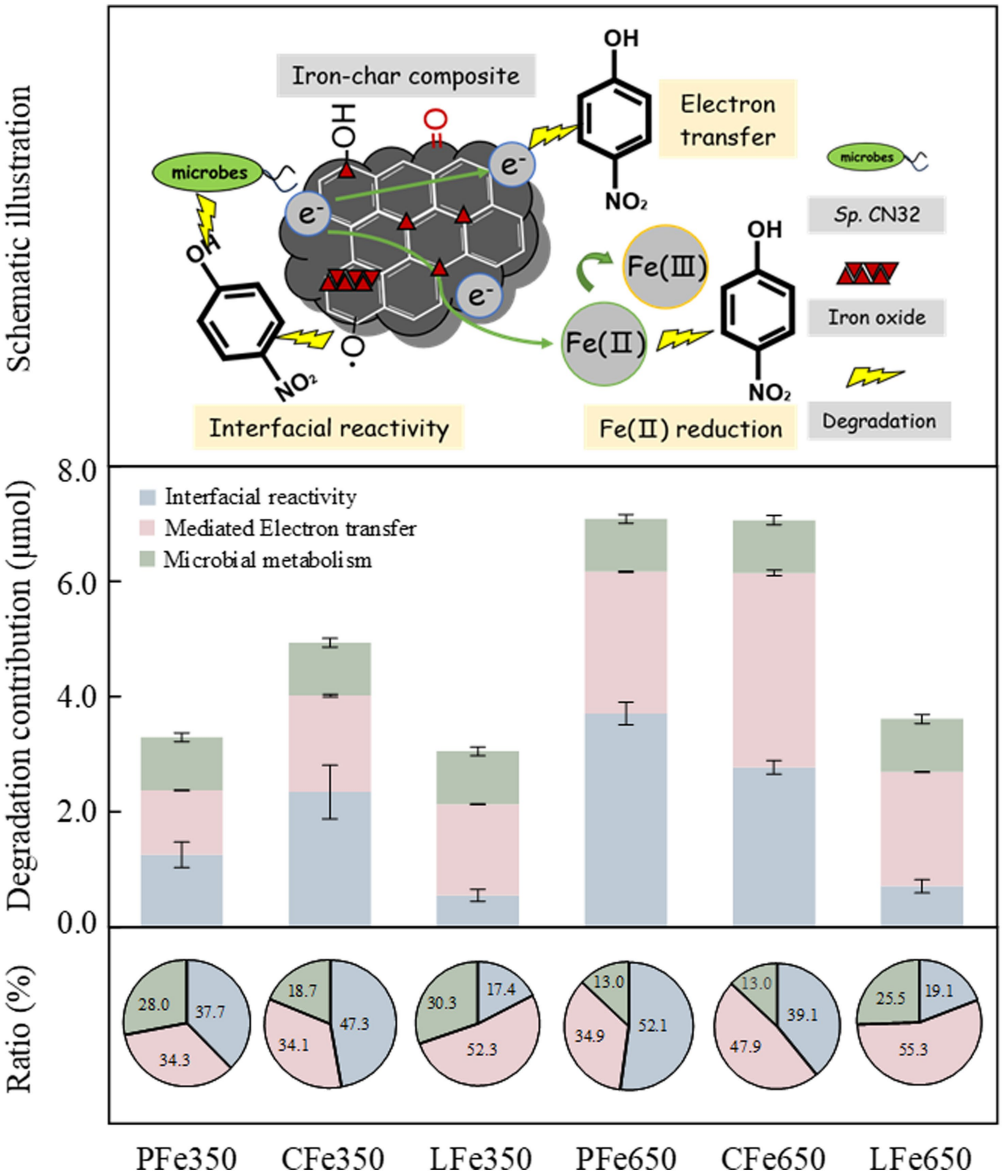
The schematic illustration and degraded contribution/ratio of iron-char composites facilitating microbial transformation of PNP in the solid and liquid phases. Three degraded pathways of iron-char-mediated PNP degradation by *Sp.* CN32 under anaerobic condition were concluded including microbial metabolism, interfacial reactivity, and mediated electron transfer. Microbial metabolism: the metabolic removal of PNP by *Sp.* CN32 alone without any iron-char composites. Interfacial reactivity: the degraded amount of PNP on the iron-char composite in the absence of *Sp.* CN32 through extraction quantification. Mediated electron transfer: the degraded amount was calculated by subtracting iron-chars-mediated PNP degradation without *Sp.* CN32 systems from those with *Sp.* CN32 systems.

Figure 8 illustrates the degradation contribution of PNP in anaerobic microbial system for the iron-char composite application. The iron-char composites consisted mainly of organic carbon matrix and inorganic iron oxides. Supplementary Figure S6 demonstrated the iron-char composite-mediated microbial transformations of PNP under anaerobic condition. The PNP degradation by the iron-char composites is synergistically controlled by microbial metabolism, interfacial reactivity, and mediated electron transfer. Obviously, it was observed that PNP degradation was limited through microbial metabolism without iron-char composites. But, after the addition of iron-char composites of low and high temperatures, PNP degradation was distinctly enhanced by 34.1–52.3% and 34.9–55.3% through the mediated electron transfer process including released Fe(II) reduction, respectively. Moreover, the iron-char composites of high temperature exhibited more superior performance for mediating extracellular electron to those of low temperature, which was attributed to more graphitic carbon and conductive magnetite, particularly in CFe650 and PFe650.

On the other hand, the interfacial reactivity of iron-char composites should not be ignored as illustrated by contribution ratio. Supplementary Figure S7 displays the existence of persistent free radicals on carbonaceous interface, suggesting iron-char composites may possess a redox activity to PNP molecules in the absence of *Sp.* CN32. As demonstrated by our previous study (Zhao et al., 2023), persistent free radicals can serve as key reactive sites for interfacial degradation. Based on the calculation of interfacial reactivity in the non-microbe system, the interfacial degradation of PFe650, CFe650, and LFe650 accounted for 52.1, 39.1, and 19.1%, and that of PFe350, CFe350, and LFe350 accounted for 37.7, 47.3, and 17.4% of the total, respectively. The degraded amount on the cellulose-derived iron-chars was obviously larger than that on the lignin-derived iron-chars, which was associated with the splendid sorption of more PNP molecules on cellulose-derived iron-chars. Nevertheless, the contribution of interfacial reactivity could be overestimated based on the calculation of PNP degradation in absence of *Sp.* CN32 system. This is because of a high probable shielding of reactive sites (e.g., persistent free radicals) on carbonaceous interface owing to electron transfer or microbial activities. Therefore, the interfacial degraded amount driven by mediated electron transfer could be still underestimated. In summary, a comprehensive understanding of the contribution of PNP degradation through the extracellular electron transfer process is essential for the optimization and application of iron-char composites in phenolic wastewater treatment in future. However, the effects of intermediate products of phenolic wastewater treatment on microbial behavior need to be further studied in future work. In addition, this study did not explore the long-term utilization potential of iron-char composites, which may constrain the applicability of this technology in nitro-phenolic wastewater treatment.

## 4 Conclusion

The role of hematite on carbonaceous structure evolution was explored via co-pyrolysis of biomass. With the rising temperature, an increase in crystalline magnetite was observed, thereby improving the magnetization of iron-char composites. The Fe 2p XPS spectra strongly revealed the increased Fe(II) species in iron-char composites during anoxic pyrolysis. The formation of C − O − Fe bond demonstrated the transformation of Fe species from inorganic to organic combined states as per O 1 s spectra deconvolution. The aromaticity and conductivity of iron-char composites were significantly enhanced with structural evolution of high-temperature. This study elucidated that iron-char composites enhanced microbial PNP degradation in anaerobic systems through facilitating mediated electron transfer, with a quantitative contribution of 34.1–55.3%. In anaerobic system with *Sp.* CN32, Fe(II) dissolution was also identified that contributed to PNP degradation. The iron-char composites of high temperature were more conducive to PNP degradation. This work evaluated the contribution of microbial transformation of PNP through application of iron-char composites and recognized the comprehensive mechanisms including microbial metabolism, mediated electron transfer, released Fe(II) reduction, and interfacial reactivity. The findings of this work will offer a bioaugmentation strategy for the efficient purification of recalcitrant nitro-phenolic wastewater.

## Data Availability

The original contributions presented in the study are included in the article/Supplementary material, further inquiries can be directed to the corresponding authors.
