# Supplementary material for: Quantitative contribution of iron-char composites mediating enhanced abatement on p-nitrophenol through extracellular electron transfer
Source: Front Microbiol. 2026 Jun 10;17:1855444. doi: 10.3389/fmicb.2026.1855444 (PMC13291006; doi:10.3389/fmicb.2026.1855444)
Supplement: Supplementary file 1 [file Table_1.DOCX]

Supplementary Material

# Supplementary Figures


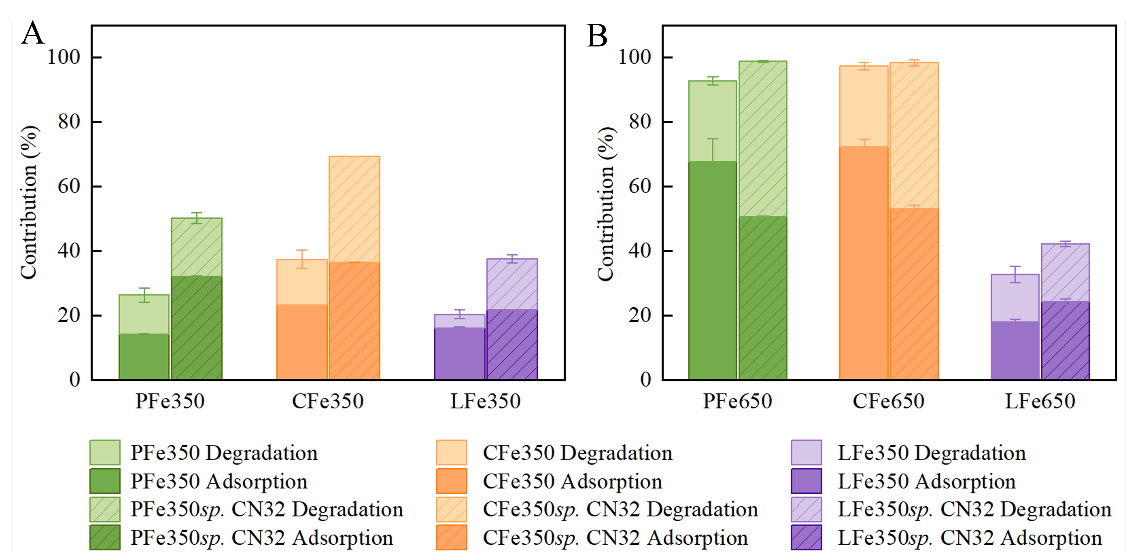


**Figure S1** The PNP removal including adsorption and degradation by iron-char composites at 350 ℃ **(A)** and 650 ℃ **(B)** within and without *Sp.* CN32 systems.





**Figure S2** The PNP removal including adsorption and degradation by iron-char composites at 350 ℃ **(A)** and 650 ℃ **(B)** through three cyclic studies.





**Figure S3** The XPS spectra of C 1s of the iron-char composites at 350 ℃ **(A)** and 650 ℃ **(B)**.





**Figure S4** Fe(II) dissolution kinetics **(A)** and total Fe concentration **(B)** of magnetite/hematite within and without *Sp.* CN32 systems.





**Figure S5** The released amount of Fe(II) in the absence and presence of PNP by iron-char composites at 350 ℃ **(A, B,C)** and 650 ℃ **(D, E, F)** in anaerobic microbial system.





**Figure S6** The ion fragments of products by iron-char composites-mediated microbial degradation using HPLC-MS.





**Figure S7** The signal intensity of persistent free radicals of iron-char composites produced from pine **(A)**, cellulose **(B)**, and lignin **(C)** at 350 ℃and 650 ℃.

# Supplementary Tables

**Table S1** Comparasion of degradation of organic contaminants by iron-based materials and microorganisms.

| Raw materials | Bacteria | The amount of iron materials used | Method | Contaminant | Removal capacity and time | Reference |
| --- | --- | --- | --- | --- | --- | --- |
| Corn stalks;  Fe(NO_3_)_3_•9H_2_O. | *Acinetobacter lwoffii* DNS32 | 95 g/L | FeMBC was fabricated via chemical co-precipitation of Fe^3+^ onto BC. | Atrazine | 3 mg;12 h | (Tao et al., 2019) |
| Aged-nZVI; aged-mmZVI. | *Shewanella putrefaciens* | 4 g/L for nZVI ; 40 g/L for mmZVI | ZVI was passivated by high concen-tration of trichloroethylene. | Trichloroethylene  (TCE) | 2.34 mg for aged-nZVI; 2.61 mg for aged-mmZVI; 20 day | (Yang et al., 2017) |
| Nano-biochars and FeCl_3_. | *Shewanella oneidensis* MR-1 | 2 mmol/L | Add both nano-biochars and FeCl_3_ to the reaction system simultaneously. | Sulfamethoxazole  (SMX) | 0.4 mg; 72 h | (Li et al., 2024) |
| Pine sawdust, cellulose, and lignin; hematite and goethite. | *Shewanella putrefaciens* CN32 | 0.6 g/L | Pyrolysis of biomass under nitrogen atmosphere(350 and 650 ℃）Add both biochar and iron oxides to the reaction system simultaneously. | *p*-nitrophenol (PNP) | 0.15-1.5 mg;7 day | (Chu et al., 2023) |

**Table S2** The fitting results using dual-stage first order kinetics model in the absence and presence of *Sp.* CN32.

| Samples | *k* _fast_ | *k* _slow_ | *f*_1_ | *f*_2_ | *f*_1_/*f*_2_ | *r*_adj_^2^ | SEE |
| --- | --- | --- | --- | --- | --- | --- | --- |
| PFe350 | 1.85 | 1.30E-03 | 0.07 | 0.93 | 0.08 | 0.98 | 0.01 |
| PFe350sp | 0.17 | 2.80E-03 | 0.21 | 0.79 | 0.27 | 0.99 | 0.01 |
| CFe350 | 0.16 | 2.50E-03 | 0.35 | 0.65 | 0.53 | 0.99 | 0.02 |
| CFe350sp | 0.11 | 2.60E-03 | 0.53 | 0.47 | 1.11 | 1.00 | 0.01 |
| LFe350 | 0.04 | 7.26E-05 | 0.29 | 0.72 | 0.40 | 0.97 | 0.02 |
| LFe350sp | 0.18 | 7.00E-04 | 0.38 | 0.62 | 0.61 | 1.00 | 0.01 |
| PFe650 | 3.61 | 3.33E-02 | 0.48 | 0.52 | 0.91 | 0.99 | 0.03 |
| PFe650sp | 6.87 | 3.61E-02 | 0.50 | 0.50 | 1.00 | 1.00 | 0.02 |
| CFe650 | 0.14 | 3.33E-02 | 0.90 | 0.10 | 8.59 | 1.00 | 0.02 |
| CFe650sp | 0.14 | 2.17E-02 | 0.90 | 0.10 | 8.83 | 1.00 | 0.02 |
| LFe650 | 0.14 | 1.00E-03 | 0.19 | 0.81 | 0.23 | 0.98 | 0.01 |
| LFe650sp | 0.19 | 5.00E-04 | 0.41 | 0.59 | 0.69 | 0.99 | 0.01 |

^a^ SEE refers to the standard error of estimate.

**Table S3** The calculated parameters as per the Tafel plots.

| Samples | Linear fitting equation of  cathode (*r*^2^) | Linear fitting equation of  anode (*r*^2^) | 10^2^I_0_ (mA) | Intersection |
| --- | --- | --- | --- | --- |
|  |  |  |  |  |
| Control | Y=-4.43749x-2.47919(0.9904) | Y=5.9088x-7.19785(0.9941) | 3.178 | (0.45, -4.48) |
| PFe350 | Y=-5.8498x-1.9722(0.9941) | Y=4.39312x-6.32474(0.9904) | 3.47 | (0.43, -4.46) |
| PFe650 | Y=-6.88646x-1.71606(0.9957) | Y=4.12152x-5.62776(0.9912) | 6.871 | (0.36, -4.16) |
| CFe350 | Y=-7.2026x-1.55934(0.9981) | Y=5.46494x-6.41865(0.9953) | 4.739 | (0.39, -4.32) |
| CFe650 | Y=-5.78815x-2.37193(0.9958) | Y=4.39228x-5.74633(0.9934) | 5.214 | (0.33, -4.28) |
| LFe350 | Y=-9.6514x-0.25202 (0.9979) | Y=7.33424x-7.70155(0.9976) | 3.243 | (0.44, -4.49) |
| LFe650 | Y=-7.2026x-1.41529(0.9981) | Y=5.46494x-6.52795(0.9953) | 4.761 | (0.40, -4.32) |

**Table S4** The deconvolution ratio of XPS spectra of Fe 2p.

| Samples | Fe^2+^ 2p_3/2_ (%) | Fe^3+^ 2p_3/2_ (%) | Sat Fe^3+^ (%) | Fe^2+^ 2p_1/2_ (%) | Fe^3+^ 2p_1/2_ (%) |
| --- | --- | --- | --- | --- | --- |
| PFe350 | 14.55 | 45.02 | 8.43 | 6.47 | 25.53 |
| CFe350 | 15.58 | 46.97 | 6.16 | 7.84 | 23.45 |
| LFe350 | 16.34 | 46.53 | 7.30 | 9.48 | 20.35 |
| PFe650 | 19.07 | 40.73 | 10.00 | 9.61 | 20.59 |
| CFe650 | 18.49 | 37.79 | 16.09 | 11.06 | 16.57 |
| LFe650 | 20.88 | 42.54 | 6.40 | 9.19 | 20.99 |

**Table S5** Elemental compositions determined by the elemental analyzer and surface element content determined by XPS.

| Sample | Elemental composition (%) | | | | |  |  | Surface elemental content by XPS (%) | | | | |
| --- | --- | --- | --- | --- | --- | --- | --- | --- | --- | --- | --- | --- |
|  | C | O | H | N | S | O/C^a^ | H/C^a^ | C | O | N | S | Fe |
| PS | 45.68 | 45.54 | 5.88 | 0.23 | 0.07 | 0.75 | 1.55 | **\** | **\** | **\** | **\** | **\** |
| CE | 41.71 | 51.77 | 6.67 | 0 | 0 | 0.93 | 1.92 | **\** | **\** | **\** | **\** | **\** |
| LI | 64.49 | 29.08 | 6.32 | 0.19 | 0.66 | 0.34 | 1.18 | **\** | **\** | **\** | **\** | **\** |
| PFe350 | 64.8 | 18.38 | 3.34 | 0.29 | 0 | 0.21 | 0.62 | 76.4 | 22.58 | 0 | 0.29 | 0.74 |
| CFe350 | 57.89 | 20.48 | 2.91 | 0.04 | 0.03 | 0.27 | 0.6 | 73.93 | 25.08 | 0.51 | 0 | 0.48 |
| LFe350 | 48.24 | 20.85 | 2.67 | 1.34 | 1.09 | 0.32 | 0.66 | 74.5 | 21.46 | 2.62 | 0.39 | 1.02 |
| PFe650 | 64.73 | 7.2 | 1.17 | 0.19 | 0 | 0.08 | 0.22 | 93.2 | 6.69 | 0 | 0 | 0.11 |
| CFe650 | 65.5 | 9.57 | 1.22 | 0 | 0 | 0.11 | 0.22 | 92.02 | 7.94 | 0 | 0 | 0.05 |
| LFe650 | 62.52 | 18.4 | 2.88 | 1.48 | 1.49 | 0.22 | 0.55 | 73.17 | 21.82 | 2.25 | 0.4 | 2.36 |

^a^ O/C and H/C refer to the polarity and aromaticity, respectively.

**Table S6** The deconvolution ratio of XPS spectra of C 1s.

| Sample | C−C(%) | C−O(%) | C=O(%) |
| --- | --- | --- | --- |
| LFe350 | 58.9 | 30.59 | 10.51 |
| PFe350 | 64.81 | 27.17 | 8.02 |
| CFe350 | 61.03 | 29.35 | 9.62 |
| LFe650 | 61.62 | 28.47 | 9.91 |
| PFe650 | 73.09 | 21 | 5.91 |
| CFe650 | 68.17 | 23.84 | 7.99 |

**Table S7** The deconvolution ratio of XPS spectra of O 1s.

| Samples | Fe−O(%) | C-O-Fe(%) | C=O(%) | C−O(%) |
| --- | --- | --- | --- | --- |
| LFe350 | 19.66 | 12.55 | 33.17 | 34.62 |
| PFe350 | 19.33 | 11.77 | 28.45 | 40.45 |
| CFe350 | 19.54 | 17.49 | 28.88 | 34.08 |
| LFe650 | 9.43 | 24.49 | 36.54 | 29.53 |
| PFe650 | 17.70 | 26.18 | 37.01 | 19.11 |
| CFe650 | 9.25 | 22.72 | 44.19 | 23.84 |

# Supplementary Text

**Text S1** Kinetic model description

The first order kinetics model of two-compartment was applied for kinetics study. The two-compartment model:

$\frac{C_{\text{t}}}{C_{0}}=f_{1}\cdot e^{-k_{1}\cdot t}+f_{2}\cdot e^{-k_{2}\cdot t}$ (1)

where *C*_0_ (mg/L) and *C*_t_ (mg/L) are the concentrations at point-in-time t and initial moment, respectively. *k*_1_ and *k*_2_ are the reaction rates of fast and slow compartments, respectively; *f*_1_ and *f*_2_ represent the relative contribution of two compartments.

Chu, Gang, Wangmin Wang, Yang Dou, Kai Sun, Wenxiu Qin, Zhen Wang, et al. (2023). Enhanced microbial degradation mediated by pyrogenic carbon toward p-nitrophenol: Role of carbon structures and iron minerals. *Science Of the Total Environment* 900. doi: 10.1016/j.scitotenv.2023.165797.

Li, Peiwen, Qiansheng Li, Hong Lu, Ze Fu, Jiti Zhou, Chenghao Sun, et al. (2024). Effect of sludge humic acid-derived nano-biochars on anaerobic degradation of sulfamethoxazole by *Shewanella oneidensis* MR-1. *Environmental Research* 251. doi: 10.1016/j.envres.2024.118655.

Tao, Yue, Songbo Hu, Siyue Han, Hongtao Shi, Yang Yang, Hanxu Li, et al. (2019). Efficient removal of atrazine by iron-modified biochar loaded *Acinetobacter lwoffii* DNS32. *Science Of the Total Environment* 682:59-69. doi: 10.1016/j.scitotenv.2019.05.134.

Yang, Zhilong, Xiao-li Wang, Hui Li, Jie Yang, Li-Yang Zhou, and Yong-di Liu. (2017). Re-activation of aged-ZVI by iron-reducing bacterium *Shewanella putrefaciens* for enhanced reductive dechlorination of trichloroethylene. *Journal Of Chemical Technology And Biotechnology* 92 (10):2642-49. doi: 10.1002/jctb.5284.

**
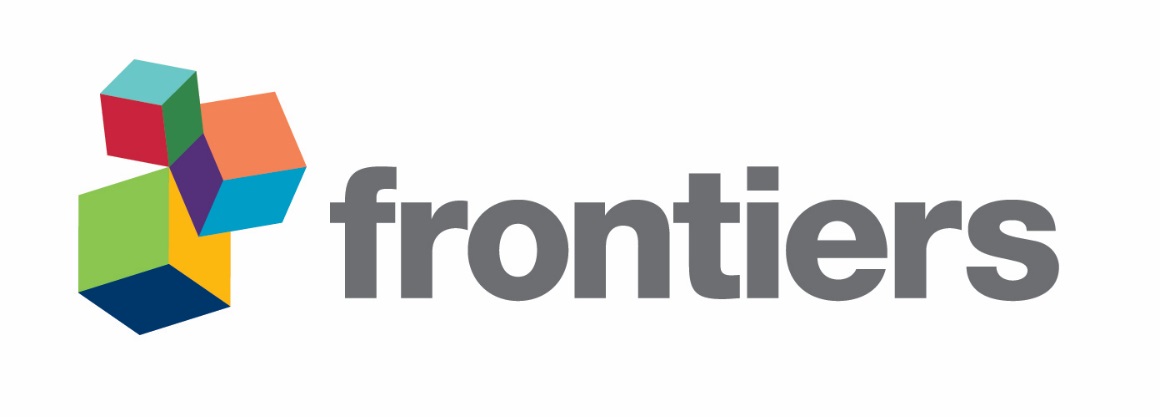
**
